# Feeding strategy and feed protein level affect the gut microbiota of common carp (*Cyprinus carpio*)

**DOI:** 10.1111/1758-2229.13262

**Published:** 2024-05-09

**Authors:** Wouter Mes, Sebastian Lücker, Mike S. M. Jetten, Henk Siepel, Marnix Gorissen, Maartje A. H. J. van Kessel

**Affiliations:** ^1^ Department of Microbiology, Radboud Institute for Biological and Environmental Sciences Radboud University Nijmegen The Netherlands; ^2^ Department of Plant and Animal Biology, Radboud Institute for Biological and Environmental Sciences Radboud University Nijmegen The Netherlands

## Abstract

Common carp (*Cyprinus carpio*) were fed food with different protein concentrations following different feeding regimes, which were previously shown to affect growth, nitrogen excretion and amino acid catabolism. 16S rRNA gene amplicon sequencing was performed to investigate the gut microbiota of these fish. Lower dietary protein content increased microbial richness, while the combination of demand feeding and dietary protein content affected the composition of the gut microbiota. Hepatic glutamate dehydrogenase (GDH) activity was correlated to the composition of the gut microbiota in all dietary treatments. We found that demand‐fed carp fed a diet containing 39% protein had a significantly higher abundance of *Beijerinckiaceae* compared to other dietary groups. Network analysis identified this family and two *Rhizobiales* families as hubs in the microbial association network. In demand‐fed carp, the microbial association network had significantly fewer connections than in batch‐fed carp. In contrast to the large effects of the feeding regime and protein content of the food on growth and nitrogen metabolism, it had only limited effects on gut microbiota composition. However, correlations between gut microbiota composition and liver GDH activity showed that host physiology and gut microbiota are connected, which warrants functional studies into the role of the gut microbiota in fish physiology.

## Graphical abstract

Common carp (*Cyprinus carpio*) were fed food with varying protein concentrations following different feeding regimes, which affected growth, nitrogen excretion and amino acid catabolism. Here, we show that host physiology and gut microbiota are connected, as these different feeding regimes have an effect on the gut microbiota.

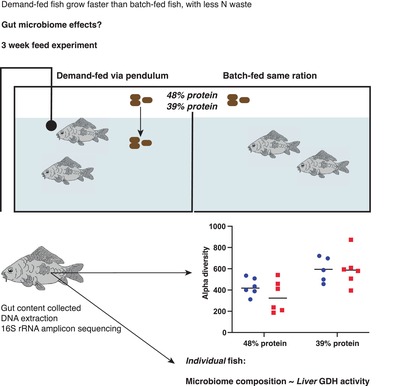

## INTRODUCTION

Aquaculture is a rapidly growing sector, last year reaching a record output of 87.5 t (FAO, 2022). As feed is a significant portion of running costs in aquaculture, optimising feed rations to obtain high growth while keeping waste as low as possible is a primary goal. The most expensive and critical component of fish feed in terms of fish growth and waste production is protein (Watanabe, 2002). If protein is used efficiently for growth, amino acids are assimilated rather than used as an energy source. This can benefit fish growth while reducing the amount of toxic ammonia excreted by the fish (Cho & Bureau, 2001). One strategy to optimise feed utilisation that has been advanced in fish aquaculture is to use so‐called demand‐feeding regimes. By synchronising the appetite of fish with the availability of feed, less feed will be wasted. This strategy has been shown to improve growth and feed conversion ratios in aquaculture settings (reviewed by Attia et al., 2012).

Demand feeding has effects on multiple physiological parameters in fish. Feeding fish according to their own biorhythm was found to increase growth in multiple species, while decreasing plasma cortisol levels and fin damage, indicating improved welfare in the animals (Azzaydi et al., 1998; Klaren et al., 2013; Noble et al., 2007, 2008). In some studies, the amount of ammonia excreted as a result of protein metabolism was also lower in demand‐fed fish compared to hand‐fed animals (Godoy‐Olmos et al., 2022; Pedrosa et al., 2019; van Kessel et al., 2016). Hence, demand feeding can improve growth and reduce nitrogenous waste production of fish at the same time.

While demand‐feeding strategies have been investigated with regard to fish growth and welfare aspects, their potential effects on the gut microbiota have not been studied. Impacts of demand feeding on the gut microbiota are likely since previous studies have found significant and rapid effects of feed uptake in general on the microbiota (Mente et al., 2018; Parris et al., 2019). In both studies, consumption of a single meal led to significant changes in gut microbial composition within hours as either a result of ingesting feed‐associated microorganisms or the growth of resident microbes in the gut. Additionally, changes in feeding regime affect gut microbial composition in other animals too (Thaiss et al., 2014). Fish intestines are colonised by a complex microbiota that is considered, as in other organisms, an additional ‘organ’ for the host, which is involved in diverse physiological processes ranging from digestion, providing vitamins, increased immunity and even influencing behaviour (Bruckner et al., 2022; Ghanbari et al., 2015; Kelly & Salinas, 2017). To understand the potential effects of demand feeding from an organismal perspective, it is key to study the interactions between host physiology and the gut microbiota.

Recent next‐generation sequencing studies of fish gut microbiomes identified several bacterial taxa present in many fish species, including *Fusobacteria*, *Proteobacteria* and *Firmicutes* (Ghanbari et al., 2015; Legrand et al., 2019). However, the relationship between microbiota composition and dietary factors, feeding regimes and physiological parameters are just beginning to be explored in fish. Furthermore, a functional understanding of the gut microbiota is still largely lacking (Ghanbari et al., 2015). Keeping in mind the effects of demand feeding on growth and nitrogen metabolism, it is particularly interesting to study the possible role of the gut microbiota in these aspects of host physiology. A recent study showed that the gut microbiota of carnivorous and herbivorous fish contributes to nitrogen metabolism to a different extent (Turner & Bucking, 2019) and a shotgun metagenomic study of the rainbow trout (*Oncorhynchus mykiss*, Walbaum) gut detected many bacterial genes involved in amino acid metabolism that may influence the utilisation of dietary nitrogen in these animals. In particular, the inclusion of yeast extract in feed led to a two‐fold increase in microbial glutamate dehydrogenase (GDH) gene clusters (Betiku et al., 2018). Beyond these findings, little is known regarding the direct effects of the gut microbiota on fish growth or nitrogen metabolism.

Here, we investigated the effects of a feeding experiment (batch‐ and demand‐feeding at two dietary protein levels, 39% and 48% crude protein, as described in Mes et al., 2023) on the gut microbiota in terms of diversity and differential abundances of specific microorganisms, and on the overall microbial association networks. The diets and feeding strategies were chosen to study the effects on nitrogen metabolism and symbiotic nitrogen cycle bacteria of carp, as described in the aforementioned study. Individual tagging of the fish allowed us to investigate how physiological and growth parameters (hepatic and intestinal GDH activities and specific growth rates) are correlated to the gut microbiota to further shed light on the role of the gut microbiota in growth and the nitrogen metabolism of carp. We hypothesised that feeding strategy has an effect on diversity indices (i.e., a different composition of the gut microbiota between demand‐fed and batch‐fed animals), while the effect of dietary protein level is predicted to be lower. The different availability of nutrients in the gut as a result of the feeding strategy is hypothesised to lead to a change in microbial associations.

## EXPERIMENTAL PROCEDURES

### Animals and feed experiment

All animal experiments were approved by the Animal Ethics Committee of Radboud University (DEC 2019‐0018, AVD1030020198606). All procedures applied to the animals were in line with the Dutch legislation (Act on Animal Experiments). In brief, juvenile common carp of both sexes (*Cyprinus carpio*, L., strain R3xF8, *n* = 120, mean body mass 16 ± 10 g) were used in a 21‐day feeding experiment, which was performed with three experimental replicates (*N* = 3). Carp were individually identified by passive integrated transponder tags (Nonatec™, Lutronic International, Rodange, Luxembourg) so individual growth rates and physiological parameters could be recorded. Fish were sampled at the end of the experiment, as described in detail in our earlier publication (Mes et al., 2023). Carp were divided into four groups based on the combination of feeding strategy (demand‐fed vs. batch‐fed) and dietary protein level (39% vs. 48% protein). The composition of each diet can be found in Table 1. Demand‐fed animals were able to feed ad libitum using a pendulum‐connected feed dispenser, in a similar setup as described in detail in Klaren et al. (2013). Batch‐fed groups of fish received the same amount of feed as the demand‐fed group consumed the day before. At the end of the experiment, fish were weighed to assess growth rates and then samples were taken from the liver, proximal intestines and the remaining intestinal sample was used to collect the gut content. The entire gut content was collected by squeezing the dissected intestines from front to back. All samples were snap‐frozen in liquid nitrogen and stored at −20°C until analysis. GDH activity rates in the liver and proximal intestine were measured as described and reported in Mes et al. (2023) and were used here to establish potential correlations with gut microbial composition. From each of the four dietary combinations, six carp were used for analysing the gut content microbial composition.

**TABLE 1:** Composition of experimental diets used in the feeding experiment.

Ingredients	39% Protein diet (g kg^−1^ ww)	48% Protein diet (g kg^−1^ ww)
Fishmeal	251	340
Soybean meal	181	181
Full‐fat extruded soybean	50	30
Fish hydrolysate	25	25
Brewer's yeast	50	50
Wheat flour	166	166
Wheat middling	153	99
Haemoglobin powder	15	5
Lecithin	5	5
Fish oil	10	10
Soybean oil	10	10
α‐Cellulose	55	0
Ca(HPO_4_)_2_	20	20
Vitamin/mineral premix	10	10
Analysed nutrient content		
Dry matter	938	949
Crude protein	392	479
Crude lipid	55	53
Crude ash	88	100
Gross energy (MJ kg^−1^)	1	19

*Note*: Vitamin/mineral premix composition (mg kg^−1^ feed): iron (as FeSO_4_ · 7H_2_O), 50; zinc (as ZnSO_4_·7H_2_O), 100; cobalt (as CoSO_4_ · 7H_2_O), 2.4; copper (as CuSO_4_ · 5H_2_O), 5; selenium (as Na_2_SeO_3_), 1; manganese (as MnSO_4_ · 4H_2_O), 25; magnesium (as MgSO_4_ · 7H_2_O), 300; chromium (as CrCl_3_ · 6H_2_O), 1; iodine (as CaIO_3_ · 6H_2_O), 5; vitamin premix composition (mg kg^−1^ feed): thiamin, 30; riboflavin, 30; nicotinic acid, 200; pantothenic acid, 100; pyridoxine, 30; cyanocobalamin, 0.05; ascorbic acid, 500; alpha‐tocopheryl acetate, 200 IU; folic acid, 15; retinylacetate, 15,000 IU; cholecalciferol, 2000 IU; menadione nicotinamide bisulphite (51%), 8; inositol, 200; choline (as choline chloride), 1000; anti‐oxidant butylhydroxytoluene (BHT) (E300‐321), 100; calcium propionate, 1000.

### DNA extraction

DNA was extracted from the complete gut content (1.2 ± 0.5 g) using the cetyltrimethylammoniumbromide‐based extraction method (Zhou et al., 1996). Briefly, samples were incubated in extraction buffer containing 10 mg mL^−1^ proteinase K at 37°C for 30 min, followed by the addition of (sodium dodecyl sulfate, 10%) and incubation for 2 h at 65°C. DNA was isolated by chloroform extraction and isopropanol precipitation and subsequently resuspended in MilliQ H_2_O. RNA was removed by RNase A treatment. Then, DNA was re‐extracted using phenol/chloroform extraction and precipitation in 70% ethanol. DNA integrity and quality were checked on a 1% agarose gel and Nanodrop. Samples with sufficient DNA concentrations (>10 ng μL^−1^) were used for 16S rRNA gene amplicon sequencing.

### 16S rRNA gene amplicon sequencing and analysis

Sequencing libraries were constructed with the Herculase II Fusion DNA Polymerase Nextera XT Index Kit V2 (Macrogen, Seoul, South Korea). Amplicon sequencing of the V3–V4 region of the bacterial 16S rRNA gene with primers Bac341F (CCTACGGGNGGCWGCAG; Herlemann et al., 2011) and Bac806R (GGACTACHVGGGTWTCTAAT; Caporaso et al., 2011) was performed by Macrogen (Seoul, South Korea) using the Illumina platform (Illumina MiSeq, 2 × 300 bp). Sequence analysis was performed in R version 4.4.1. Raw reads were processed using the Divisive Amplicon Denoising Algorithm 2 (DADA2) pipeline (Callahan et al., 2016). Forward and reverse reads were quality‐trimmed and primers were removed, after which the DADA2 algorithm was used to infer amplicon sequencing variants (ASVs) from the processed forward and reverse reads separately using the pooled samples. The forward and reverse ASVs were merged (minimum overlap of 12 bases) to obtain full‐length amplicon ASVs. Chimeric ASVs were removed using the consensus‐based chimaera removal tool of DADA2, after which the taxonomic assignment of valid ASVs was determined using the naïve Bayesian classifier method with the SILVA SSU rRNA database (version 138) as a reference dataset (Quast et al., 2012). From the raw sequences, 771 ASVs were inferred after processing with DADA2. The inferred ASVs were aligned using the multiple alignment using the fast Fourier transform programme and a maximum likelihood tree was calculated in MEGA X (Kumar et al., 2018; Madeira et al., 2019).

### Microbiota analysis and statistical analysis

Statistical analysis of the microbiota of each sample was performed using the ‘phyloseq’ and ‘microbiome’ R packages (Lahti et al., 2017; McMurdie & Holmes, 2013). ASVs that were present in only one sample and ASVs not unambiguously assigned to a bacterial phylum were removed, as well as any ASVs that were of mitochondrial or chloroplast origin. Alpha diversity measures (Shannon diversity and Chao1 richness) were calculated from the untransformed ASV counts per sample. The effect of the feeding strategy and dietary protein level on alpha diversity of the microbiota was tested with a two‐way analysis of variance (ANOVA) in Graphpad Prism version 9.1 for Mac (Graphpad software, La Jolla, USA).

Bray–Curtis dissimilarities and weighted UniFrac distances were calculated using untransformed count data and a principal coordinate analysis was used to plot the calculated beta diversities (differences in microbial composition). The R package ‘vegan’ was used to determine if the microbial composition was statistically different between feeding strategies and dietary protein levels, and the effects of intestinal and hepatic GDH activities and specific growth rate. A permutational multivariate analysis of variance (PERMANOVA) analysis was performed with the adonis2 function with feeding strategy, dietary protein level, specific growth rate, hepatic GDH activity and intestinal GDH activity as variables and 999 permutations. Interaction terms between variables were also tested. Only the interaction between feeding strategy and dietary protein level had a significant effect and was included in further analysis for differential abundances.

The ‘ALDEx2’ package was used to identify differentially abundant microorganisms between groups. The analysis was based on a general linearised model with feeding strategy and dietary protein level as factors, with ASVs clustered on the genus and family level as input (Fernandes et al., 2014). To avoid bias in the differential analyses due to the compositional nature of the sequence data, count data were transformed using a centred log ratio (clr‐transformation). The structure of the formula was as follows: feeding strategy × dietary protein level. Differentially abundant genera (false discovery rate‐corrected *p* value <0.05) were identified. Furthermore, correlations of clr‐transformed abundance data with continuous variables (specific growth rate, hepatic and intestinal GDH activity) were also calculated using the ALDEx2 package.

We investigated correlations between the abundances of bacteria of the carp gut microbiota as well as correlations between abundances and experimental treatments. We used the ‘NetCoMi’ package to calculate microbial correlation matrices and perform network analyses on the calculated networks (Peschel et al., 2021). Microbial correlation analysis was performed on the ASV level, as well as family‐clustered counts using the SPRING method (Yoon et al., 2019) with a filtering step of presence in at least five samples in order to be included. Correlations were transformed to dissimilarities using the ‘signed’ distance metric and the similarities were used as edge weights. The size of nodes in the constructed network was scaled based on the eigenvector centrality of the node. To determine differences in microbial networks between demand‐fed and batch‐fed carp guts, the same methods were used, but with a different filtering step to only use taxa that were represented in at least 25% of the samples in both groups. These networks were constructed at the genus level, as too few families passed the filtering step to construct reliable networks. Network characteristics were compared using a permutation test (100 permutations).

## RESULTS

### Dietary protein content affects species richness of the gut microbiota

**FIGURE 1:**
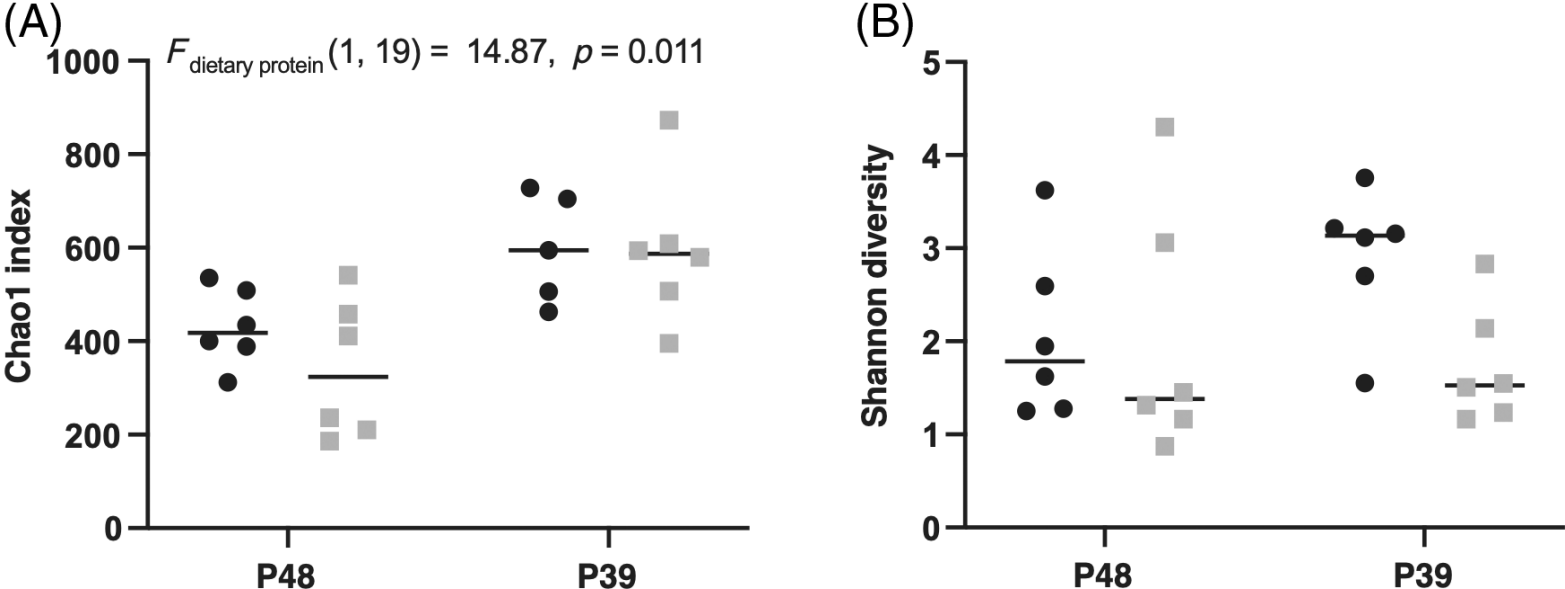
Alpha diversity of microbiota of demand‐fed (●) and batch‐fed (■) carp fed with food containing 48% and 39% protein. Each symbol corresponds to one fish, with lines indicating median values. (A) Chao1 index (ASV richness) of alpha diversity per dietary treatment. Dietary protein level had a significant effect on Chao1 diversity index values (*F* (1, 19) = 14.87, *p* = 0.0011). (B) Shannon diversity index (ASV richness and evenness) of alpha diversity per sample type.

The overall microbial richness and evenness of the gut microbiota in each experimental group were compared using the Chao1 and Shannon diversity indices (Figure 1). Higher dietary protein concentration had a significant (negative) effect on the Chao1 index (*F* (1, 19) = 14.87, *p* = 0.0011), which is exemplified by a lower number of ASVs detected in carp fed the 48% compared to the 39% protein diet, regardless of feeding strategy. In contrast, the Shannon diversity index was not significantly affected by feeding strategy or dietary protein level. These findings together indicate that protein level influences the overall number of ASVs, while the evenness was comparable between dietary groups. In other words, it is likely that the increased diversity observed in the 39% protein group is due to an increase in rare taxa.

### Dietary groups have different microbial composition

**FIGURE 2:**
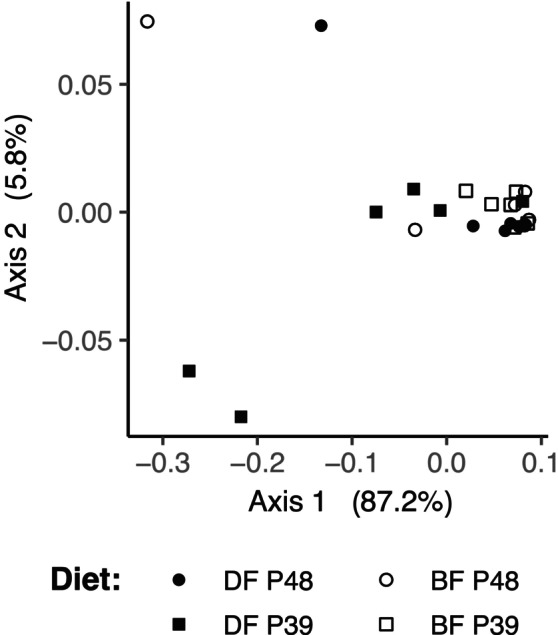
Beta diversity of microbiota of demand‐fed and batch‐fed carp gut based on weighted UniFrac distances. The different dietary treatments are indicated with different symbols: closed symbols correspond to demand‐fed (DF) carp, while open symbols correspond to batch‐fed (BF) carp, circles correspond to 48% protein diets (P48) and squares correspond to 39% protein diets (P39). The interaction between feeding strategy and dietary protein level significantly affected weighted UniFrac distances (*F* (1, 1) = 4.1708, *r* ^2^ = 0.13755, *p* = 0.038), as well as hepatic glutamate dehydrogenase activity (*F* (1,1) = 4.1297, *r* ^2^ = 0.13619, *p* = 0.047.

Feeding strategy and dietary protein level both had a measurable effect on beta diversity between gut microbiota samples. When only microbial composition was analysed (using Bray–Curtis dissimilarities), there was a significant effect of feeding strategy as well as of the combined effects of feeding strategy and dietary protein level (*F* (1, 1) = 4.1500, *p* = 0.017, and *F* (1, 1) = 3.6704, *p* = 0.040, respectively, Figure S1 and Table S1 for PERMANOVA test). However, if phylogenetic distance is taken into account by using weighted Unifrac distances (Figure 2), beta diversity was only affected by the combined effects of feeding strategy and dietary protein (*F* (1, 1) = 4.1708, *p* = 0.038). Based on the principal coordinate analysis plot, it is apparent that the gut microbiota of carp that were demand‐fed with the 39% protein diet is the most distinct from the other three groups, despite some overlap in composition (Figure 2). Furthermore, there is a significant correlation between hepatic GDH activity of carp and the weighted Unifrac distances, regardless of their dietary treatment (*F* (1, 1) = 4.1297, *p* = 0.047). The combined effect of feeding strategy and dietary protein explains approximately 14% of the observed variation, while hepatic GDH activity explains another 13% (Table S2 for PERMANOVA test overview).

**FIGURE 3:**
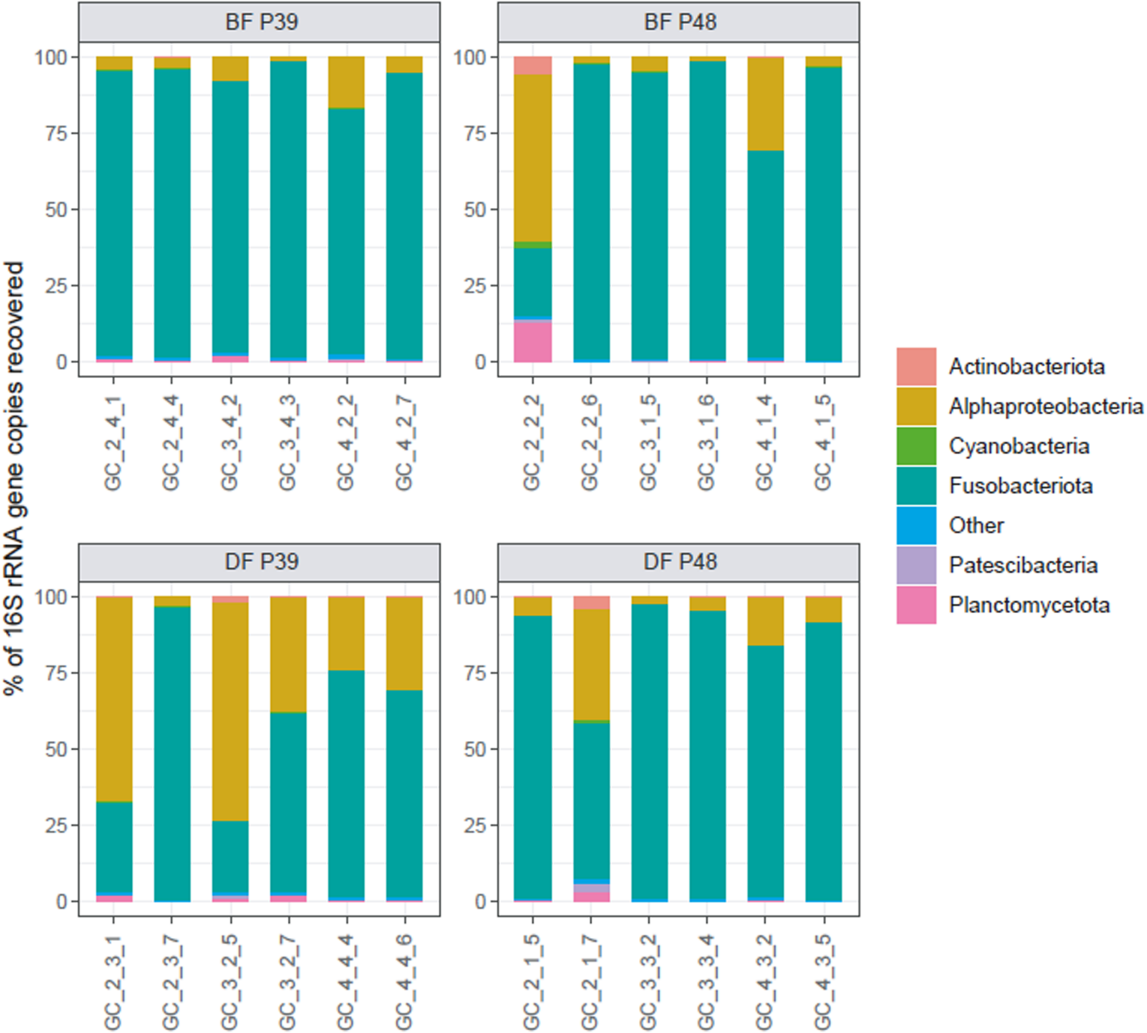
Composition of the individual carp gut microbiota of the different dietary groups. Relative abundances of major phylum‐level groupings are shown (>5% relative abundance), with phyla below this value classified as ‘Other’ and with Proteobacteria split further to class level. BF, batch‐fed; DF, demand‐fed; P39, 39% protein diet group; P48, 48% protein diet group.

The distinct clustering of the demand‐fed group fed with the 39% dietary protein diet (DF‐P39) from the other dietary treatments is further apparent if the individual composition of the microbiota on a phylum level is taken into consideration. While most of the microbiota of carp from the other groups are dominated by *Fusobacteriota* (often for more than 90% of total 16S rRNA gene sequences), the gut microbiota of DF‐P39 carp show an increased presence of *Alphaproteobacteria* (Figure 3). This pattern in the composition is visible in most DF‐P39 carp and only in single individuals of other groups.

To further identify the taxa responsible for the observed differences between the gut microbiota of carp from different dietary treatments, we performed a differential abundance analysis using the ALDEx2 algorithm, using genus‐ or family‐clustered taxa. After correcting for the false discovery rates, only a single genus was differentially abundant between DF‐P39 and the other dietary treatments, which is the alphaproteobacterial *Bosea* (*p* = 0.032). The average relative abundance of this taxon in DF‐P39 carp is >2.5%, while in the others it remains at ≤1%. The family *Beijerinckiaceae* (to which *Bosea* belongs) was differentially abundant as well (*p* = 0.024). No specific taxa were significantly correlated to the hepatic GDH activity according to the ALDEx2 algorithm, despite the significant effect on the overall microbial composition.

### Network analysis of the carp gut microbiota

While the different dietary treatments of the carp in our experiment did lead to differences in beta diversity, most taxa were not differentially abundant. This led us to investigate the effect of the dietary treatments on the microbial associations in the carp gut. The SPRING algorithm was used to construct sparse microbial association networks. First, an association network was constructed at the ASV level for the overall gut microbiota of all carp. In this network, seven modules of co‐associating ASVs were identified that were more strongly interacting within the modules than outside (Figure S2). Further, six hub ASVs were identified that have a high connectedness and importance for the overall network structure. The taxonomic placement of these six ASVs was as follows: two *Cetobacterium* ASVs and one ASV each affiliated with Ca. Megaira, *Reyranella*, *Mycobacterium* and *Fimbriiglobus*.

**FIGURE 4:**
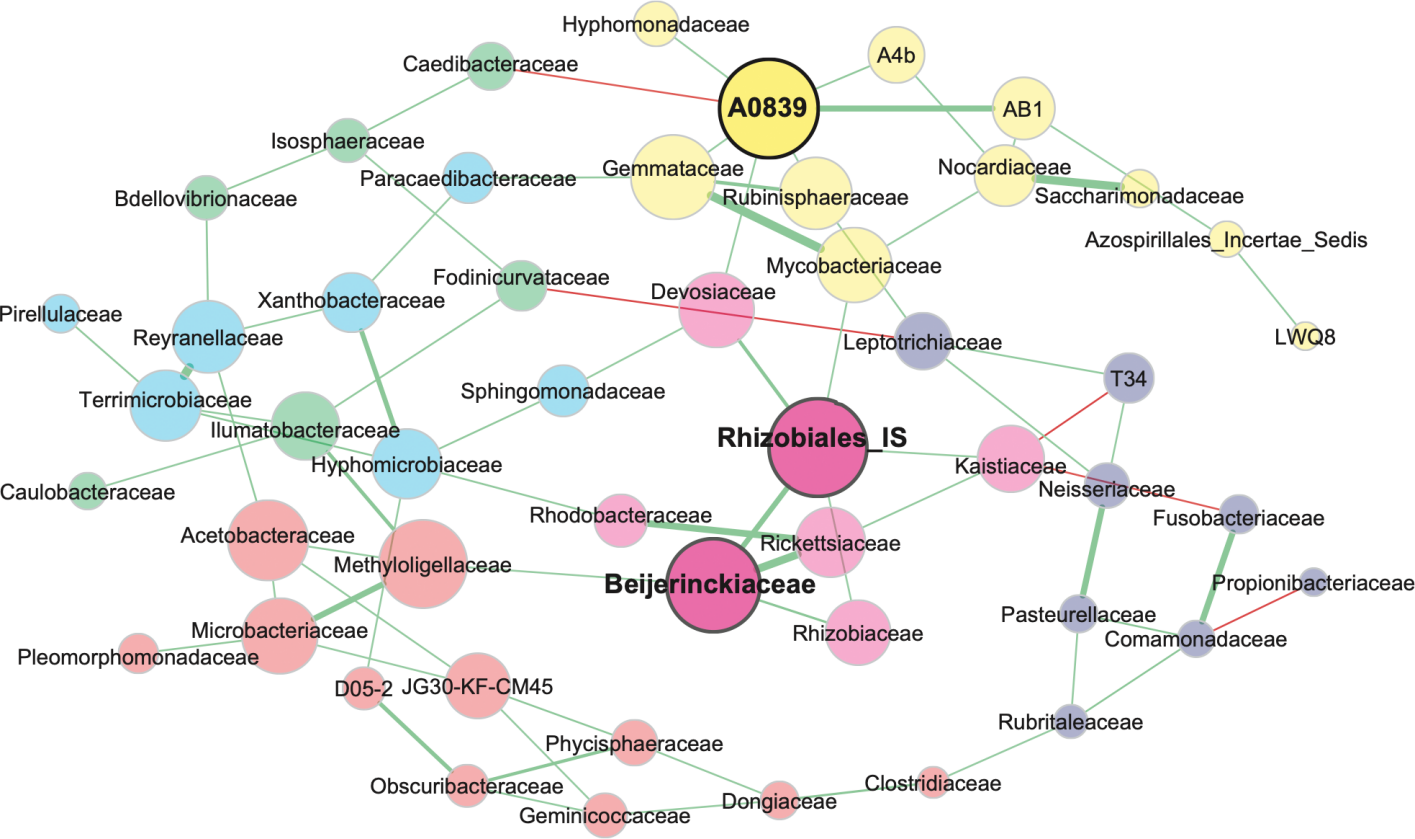
Microbial association network of the carp gut at family level. All carp gut samples (*n* = 24) were used to compute the network. Only families found in at least five samples were used for the construction of the network. Different colours indicate modules that correlate together. Family names and nodes in bold indicate hub families. Green and red connections indicate positive and negative correlations, respectively. The thickness of lines indicates the strength of the correlation.

At the family level, another association network was constructed for all gut samples together (Figure 4). This network is sparser due to the higher taxonomic level, with the number of modules reduced to six, and the number of hub families reduced to three. Still, it provided additional insights into community structure. Interestingly, the microbial correlation network suggests that the most abundant family (*Fusobacteriaceae*) was not central to the network, while less abundant alphaproteobacterial families had higher centrality values. All three hub families were *Alphaproteobacteria* from the order *Rhizobiales*, one of which was *Beijerinckiaceae* that was differentially abundant between DF‐P39 and the other dietary treatments. The other hub families are unclassified *Rhizobiales* and a family of uncultured *Rhizobiales* known as A0839.

**FIGURE 5:**
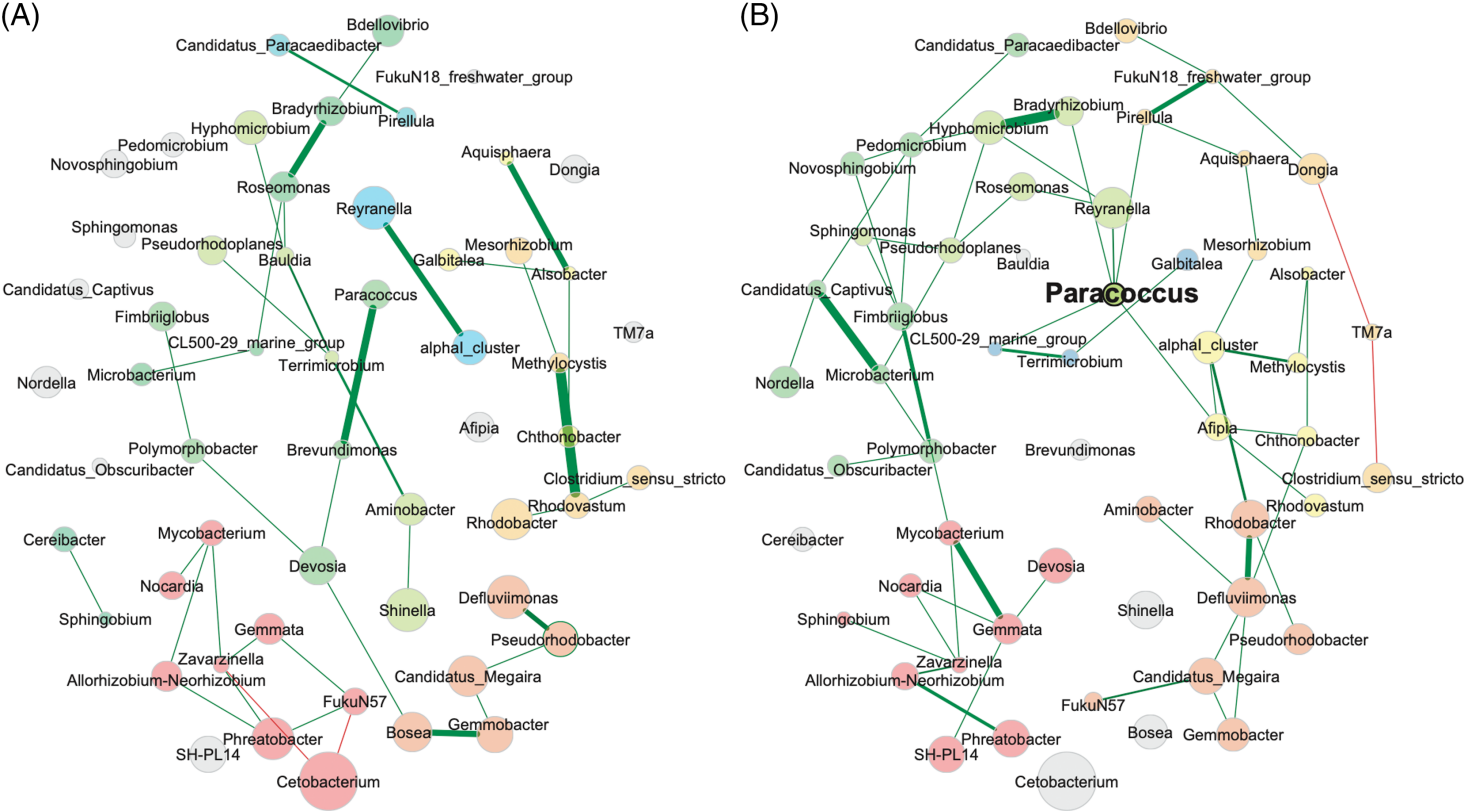
Microbial association networks of demand‐fed (A) and batch‐fed (B) carp guts at genus level. The demand‐fed and batch‐fed carp gut networks were created using samples from both 39% protein and 48% protein diets (*n* = 12) for each group. Only genera present in more than three individuals per group were used to construct the networks. Different colours indicate correlated modules, while grey nodes are unconnected genera. A genus name in bold indicates a hub genus. Green and red connections indicate positive and negative correlations, respectively. The thickness of the lines indicates the strength of the correlation.

To parse out differences between demand‐fed and batch‐fed carp, we contrasted the networks (at genus level, as at the family level no correlation network could be constructed) occurring in demand‐fed with those in batch‐fed carp gut samples to see if the centrality of specific taxa is different (Figure 5). While the overall microbial composition and diversity were not highly distinct between groups, the underlying correlation between the microbes was significantly affected. The overall network analysis showed that the associations between microbial genera are different in demand‐fed and batch‐fed animals (see Table S3 for the complete test output). Most strikingly, the overall connectedness of the network is significantly reduced in demand‐fed carp. Only 18% of the taxa are part of the largest connected component (LCC) of the network, whereas this was 82% in batch‐fed animals (*p* = 0.010), which means that more of the taxa in batch‐fed animals are part of an interconnected network. The modules inside the LCC are more distinct in batch‐fed fish (*p* = 0.020). As a result, the total network of demand‐fed carp consists of 24 separate clusters, compared to 12 in batch‐fed carp (*p* = 0.040). In line with the different network structures, the modularity of the network (how distinct clusters inside the network are) is higher in demand‐fed than batch‐fed animals (*p* = 0.040). In short, the demand‐fed carp microbial association network is less connected but composed of more distinct modules, while the network of batch‐fed carp is more connected as a whole. There are also no hub taxa in the demand‐fed carp gut network, further indicating reduced connectedness. In contrast, in batch‐fed carp most taxa are connected with one another and the genus *Paracoccus* is the single hub genus identified.

The centrality measures of nodes inside the network were all significantly different too, which further indicates that the networks as a whole are dissimilar. The clusters calculated for each network are distinct (as calculated by the Adjusted Rand Index [ARI]) and are significantly different from that of two random clustering. This indicates that the networks have some clustering in common (ARI = 0.134, *p* < 0.001), which is reduced when only the LCC is considered (ARI = 0.049, *p* = 0.001).

## DISCUSSION

Demand feeding has been shown to improve growth rates and welfare in multiple fish species (Azzaydi et al., 1998; Klaren et al., 2013; Mes et al., 2023; Noble et al., 2007, 2008). This study is, to our knowledge, the first in which the effect of feeding regime on the gut microbial community is combined with individual physiological parameters. We hypothesised that demand feeding would affect gut microbiota composition to a larger extent than dietary protein content, in line with its effects on fish physiology found in the study with the same carp (Mes et al., 2023). We additionally hypothesised that microbial associations in the gut microbiota were affected by demand feeding. We found that a higher dietary protein content decreased the richness of the gut microbiota and that demand‐fed carp had a significantly different gut microbial composition compared to batch‐fed carp, while the largest difference was observed between demand‐fed carp fed the 39% protein diet (DF‐P39) and all other groups. Hepatic GDH activity also had a small, yet significant, effect on microbial composition. The microbiota of the DF‐P39 group was characterised by a higher abundance of *Alphaproteobacteria*, with the genus *Bosea* (family *Beijerinckiaceae*) differentially abundant between the DF‐P39 carp and the other groups. Finally, microbial network analysis identified three alphaproteobacterial families as hub families that are essential to keep the overall network connected, including the differentially abundant family *Beijerinckiaceae*. Comparing the microbial co‐occurrence of demand‐fed and batch‐fed carp guts showed a reduction in connectivity in demand‐fed microbial networks.

Based on our findings, demand feeding had a relatively limited impact on the composition of the gut microbiota, as the overall similarity between groups was high. Only a single family of microorganisms (*Beijerinckiaceae*) was significantly different in abundance between DF‐P39 and the other dietary groups and no overall differentially abundant taxa were identified between the main factors (dietary protein content and feeding strategy). The combination of protein level and feeding strategy only explained 14% of the variation in composition. This is in contrast to what we hypothesised, based on the large effects demand feeding has on the growth rates and nitrogen metabolism that we previously found in the same individual fish (Mes et al., 2023). It is possible that the duration of our experiment (21 days) was not long enough to observe large‐scale alterations in the gut microbiota. Gut microbiota of other fish species were, however, found to change considerably even after being fed a single meal, so the short duration of the feeding experiment does not necessarily explain the small effect observed here (Mente et al., 2018; Parris et al., 2019). Although diet was shown to affect microbial composition of the gut in many fish species (reviewed by Tarnecki et al., 2017) there are also studies that report no or very little effect of diet (Bakke et al., 2015; Lyons et al., 2017). Even though the microbial composition between groups investigated here was largely similar, the underlying microbial associations were affected, so it may be possible that demand feeding rather affected how different microorganisms are associated to one another. Whether this leads to changes in microbial composition in the longer term requires a feed experiment with a longer duration.

Dietary protein content, by contrast, had no effect on beta diversity indices but did have a significant effect on the Chao1 richness index, with the 39% protein content groups having an increased number of ASVs present. This is in line with research showing that carnivorous fish species (which generally have a diet with higher protein concentrations) have less diverse microbiota compared to omnivorous and herbivorous species (Liu et al., 2016). Additionally, in another carp species, animals fed an animal protein‐based diet had a lower species richness than those receiving a plant protein‐based diet (Hao et al., 2017). The reason for this reduced diversity is not entirely explained, but may have to do with the higher digestibility of protein compared to other feed ingredients and a reduced capacity for cellulose digestion (Liu et al., 2016). The 39% protein diet in our study contained α‐cellulose and more wheat middling compared to the 48% protein diet, so the effects on ASV richness can also be mediated by increased cellulose or fibre content instead of solely decreased dietary protein content. It is important to consider that both diets used in our study are high in protein content for an omnivorous fish like common carp, which has an optimal dietary protein requirement of around 30%–35% (Watanabe, 1982). The choice of diets was based on the goal of comparing the effects of nitrogen cycle symbionts in the gills of carp (as described in Mes et al., 2023), and it is possible that a diet that is lower in dietary protein content can have more considerable effects on the gut microbiota.

The gut microbiota of the carp in our experiment was mostly dominated by *Fusobacteria*, particularly from the genus *Cetobacterium*, which is commonly found in the gut of freshwater fish and is known to produce vitamin B12 (Tarnecki et al., 2017; Tsuchiya et al., 2008). Compared to the first description of the gut microbiota of common carp, the relative abundance of *Cetobacterium* is considerably higher (van Kessel et al., 2011). In another carp species, *Fusobacteria* abundance was higher in fish fed with fish meal‐containing diets compared to plant protein diets (Hao et al., 2017). As fish meal made up most of the protein in both of our experimental diets and protein levels were high overall, this may explain the higher relative abundance of *Fusobacteria* in our study. The study of Hao et al. also identified a strong negative correlation between *Fusobacteria* and *Bacteroidetes*, which we observe here too. The relative abundance of *Bacteroidetes* is very low in all our carp compared to previously published literature (Hao et al., 2017; van Kessel et al., 2011). Overall, the number of phyla found in the gut microbiota of carp from our feed experiment was lower compared to that described in van Kessel et al. (2011), but in line with other common carp gut microbiota descriptions that also find *Fusobacteria* and *Proteobacteria* to make up around 80%–90% of the ASVs present (Chang et al., 2020; Eichmiller et al., 2016; Zhang et al., 2021).

The high abundance of *Alphaproteobacteria* in several carp individuals (primarily from the DF‐P39 group) is interesting as well. Previous descriptions of the carp gut microbiota have not found such high abundances (van Kessel et al., 2011; Zhang et al., 2023), although in the study by van Kessel et al., different 16S rRNA gene primers were used which may affect the observed microbiota composition. The one taxon significantly affected by feeding strategy and dietary protein was the alphaproteobacterial *Bosea*, suggesting that our dietary treatment was selected for this bacterial group. Few functional descriptions are available for microorganisms in the fish gut, but in crucian carp (*Carassius carassius*, L.) the genus *Bosea* was significantly increased in fish fed a diet enriched in short‐chain fatty acids. *Bosea* species have also been found to express an enzyme that degrades *N*‐acyl homoserine lactones (AHLs) used for quorum sensing. AHLs are used by pathogenic bacteria in the regulation of density‐dependent virulence and the disruption of such quorum sensing molecules of pathogens could therefore be beneficial for host health (Li et al., 2022; Zhang et al., 2019).

The family *Beijerinckiaceae* to which *Bosea* belongs, was identified as one of the hub families in the microbial association network. The other hub families were also *Alphaproteobacteria* from the order *Rhizobiales*. *Rhizobiales* are particularly abundant in and seem to be relevant for the formation of biofilms (Pang & Liu, 2007) and are frequently found in recirculating aquaculture system biofilter microbiomes (Sugita et al., 2005). In zebrafish (*Danio rerio*, Hamilton), gut microbiota were inversely correlated to pathogenic bacterial abundance (Chen et al., 2018). Their specific functions in carp intestines are still unknown, but their role in forming biofilms and hence influencing colonisation patterns of other bacteria would explain a central place in the microbial association network. In general, looking at microbial interactions in the gut may be more informative than only studying microbial composition.

Using network analysis as a tool in microbiota research is relatively novel, in particular in its application to fish microbiota studies (Barberán et al., 2012; Sylvain & Derome, 2017). In our study, although overall differences in composition were small, the associations between the microorganisms that make up the gut microbiota were clearly distinct in demand‐fed and batch‐fed animals. Specifically, the demand‐fed carp gut microbiota were characterised by more, but less well‐connected co‐association modules compared to the batch‐fed carp gut microbiota. The LCC of the demand‐fed network covered 18% of the total network, compared to 82% in the batch‐fed network. Although the overall connectivity was lower, clustering into distinct modules (modularity of the network) was higher for demand‐fed gut microbiota networks. Furthermore, the interactions of the taxa within the networks were also significantly different between feeding strategies, which was clear from the significant differences in centrality measures and a low Rand index value. It is thus apparent that while the overall community composition remained relatively stable in the feed experiment, the underlying interactions between microorganisms were affected. A less connected network is generally seen as a less stable situation (i.e., more sensitive to disruption) (Santolini & Barabási, 2018), which may indicate that demand feeding leads to reduced stability of the gut microbiota. However, increased modularity is also associated with a higher stability (Gao et al., 2021), which was increased in demand‐fed networks. Hence, it is not possible to draw conclusions on the effects of demand feeding on overall network stability, but this should certainly be investigated in future long‐term studies.

In our previous study using the same carp, we found significant effects of demand feeding on specific growth rates and hepatic and intestinal GDH activities (Mes et al., 2023). Here, we determined if these individual physiological parameters could explain variations in gut microbiota composition. In contrast to what we hypothesised, we did not find any significant correlation between microbial composition and growth rates. This is different from recent studies in cyprinids, in which body mass and growth rates affected gut microbiota composition (Nie et al., 2022; Wang et al., 2020). However, a lack of correlation has also been reported when fast‐ and slow‐growing rainbow trout gut microbiota were compared (Chapagain et al., 2019). However, in contrast to our study, the studies focusing on cyprinids were performed in pond culture, which may lead to different influences on the gut microbiota. Additionally, we utilised the ALDEx2 compositional differential abundance method to perform correlation analysis, which was recently found to be a more conservative test compared to the linear discriminant analysis with effect size test performed in the aforementioned studies (Nearing et al., 2022). This may have reduced the number of correlations we observed. Finally, it is also possible that while growth rates had no effect on microbial composition, the differences in microbial association found in demand‐fed and batch‐fed carp are in part correlated to the different growth rates.

As the feeding strategy was previously found to affect the nitrogen metabolism of our carp, we also investigated possible correlations between the activity of the important enzyme responsible for ammonia production from amino acids (GDH) and gut microbial composition. GDH activity is responsible for deaminating glutamate to alpha‐ketoglutarate and ammonia, and is the final step of amino acid catabolism (Ip & Chew, 2010). We predicted an effect in both intestinal and hepatic GDH activity, possibly through increased digestion and uptake of amino acids mediated by the gut microbiota. Only hepatic GDH activity explained a significant amount of variation observed in microbial composition, approximately the same amount as feeding strategy and dietary protein level (13%). The correlation of hepatic amino acid metabolism and gut microbiota composition can be explained by an increased availability of amino acids for the fish, which shifts the amino acid metabolism for protein synthesis towards oxidation of the carbon skeleton and energy conservation. It is hence associated with reduced protein efficiency in the growth of fish (Betiku et al., 2018). If this is indeed the explanation for the observed correlation, it can be that plasma amino acid concentrations will increase as well.

It is unlikely that the ammonia produced through hepatic GDH activity has affected the gut microbiota, since the overwhelming majority of ammonia produced by fish is excreted via the gills (around 90%) and the gut is not shown to be a major site of ammonia excretion from the bloodstream, although it can be a source of additional ammonia production (Bucking, 2017; Rubino et al., 2014). Also, as GDH activity in the proximal intestine had no effect on gut microbial composition, which is closer to the gut lumen, it is even less likely. Hence, it is more likely that the difference in microbiota composition can partly explain elevated hepatic GDH levels, possibly via increased amino acid uptake. Similar effects of the gut microbiota on liver functioning have recently been observed in mammals, where microorganisms in the gut play key roles in entraining metabolic circadian rhythms in the liver via metabolites such as short‐chain fatty acids and microbially modified bile acids, which were affected by feeding regime and dietary composition (Alvarez et al., 2020; Frazier & Chang, 2020). While such research has yet to be performed in fish, the role of the microbiota in entraining the circadian rhythms of host metabolism may be equally important. These findings are also important to keep in mind for studies investigating demand‐feeding effects in general: while demand feeding may affect the gut microbiota, it may also allow the gut microbiota to entrain beneficial circadian rhythms in the animals that could explain the observed effects on growth and nitrogen metabolism. A demand‐feeding setup could be used to detect the presence of microbial metabolites in the bloodstream of the host and extend our understanding of demand feeding and feeding rhythms on the organismal performance of fish.

## CONCLUSION

Our results indicate that demand feeding in combination with dietary protein content affects the microbial composition as well as microbial associations in carp intestines. A combination of demand feeding with 39% dietary protein diet led to a more diverse microbiota including a higher abundance of *Beijerinckiaceae*. This family of *Alphaproteobacteria* was also identified as a keystone taxon in the microbial association network of carp intestines. Demand feeding led to a change in microbial association patterns, with a reduction in network connectivity. Furthermore, we find that regardless of feeding strategy, hepatic GDH activity was linked to gut microbiota composition, which is an indication that the gut microbiota influences host physiology beyond the gut, possibly explaining the altered nitrogen metabolism in demand‐fed carp previously discovered. Demand feeding thus did not lead to large changes in gut microbial composition but changed the underlying association networks, which may lead to more marked changes in composition in the longer term.

## AUTHOR CONTRIBUTIONS

**Wouter Mes:** Conceptualization (equal); formal analysis (equal); investigation (equal); writing – original draft (lead). **Sebastian Lücker:** Conceptualization (equal); supervision (equal); writing – review and editing (supporting). **Mike S. M. Jetten:** Funding acquisition (equal); supervision (equal); writing – review and editing (supporting). **Henk Siepel:** Supervision (supporting); writing – review and editing (supporting). **Marnix Gorissen:** Conceptualization (equal); funding acquisition (equal); supervision (equal); writing – review and editing (equal). **Maartje A. H. J. van Kessel:** Conceptualization (equal); formal analysis (equal); investigation (equal); supervision (equal); writing – original draft (equal).

## CONFLICT OF INTEREST STATEMENT

The authors declare no competing interests.

## ETHICS STATEMENT

Animal experiments were approved by the Animal Ethics Committee of Radboud University (DEC 2019‐0018, AVD1030020198606).

## Supporting information


**Data S1.** Supporting Information.

## Data Availability

The dataset supporting the conclusions of this article is available in the SRA database under project number PRJNA987445 (https://www.ncbi.nlm.nih.gov/bioproject/PRJNA987445). GDH activities and specific growth rates of the animals are published in Mes et al. (2023).
