# Supplementary material for: Feeding strategy and feed protein level affect the gut microbiota of common carp (Cyprinus carpio)
Source: Environ Microbiol Rep. 2024 May 9;16(3):e13262. doi: 10.1111/1758-2229.13262 (PMC11082430; doi:10.1111/1758-2229.13262)
Supplement: Supplementary file 1 — Data S1. Supporting Information. [file EMI4-16-e13262-s001.docx]

**Fig. S1** **Ordination plot of carp gut microbiota samples based on Bray-Curtis dissimilarity values**.

Different dietary treatments are indicated with different symbols. Feeding strategy significantly affected Bray-Curtis dissimilarity values (F (1, 1) = 4.15, r^2^ = 0.12754, p = 0.017) as well as the interaction between feeding strategy and dietary protein level (F (1, 1) = 3.6704, r^2^ = 0.11280, p = 0.040). and hepatic glutamate dehydrogenase activity (F (1,1) = 3.7023, r^2^ = 0.11378, p = 0.037).

**Fig. S2 Microbial association network of the carp gut at amplicon sequencing variant (ASV) level.** The network was constructed using the SPRING algorithm with the NetCoMi package. Only ASVs with >1000 reads on average were used for the construction of the network. Different colors indicate different modules that correlate together. ASV numbers and nodes in bold indicate hub families that have high centrality for the overall network. Green and red edges indicate positive and negative correlations, respectively. The thickness of edges indicates the strength of the correlation.

**Table S1 PERMANOVA test on Bray-Curtis dissimilarity values.**

Each factor tested as explanatory variable for the observed Bray-Curtis dissimilarity values is shown with the degrees of freedom (Df), sum of squares (SumOf), R^2^-, F- and p-values. Factors that have a significant effect on the dissimilarity values are indicated in **bold**, with asterisks indicating the level of significance. Feeding strategy and Dietary protein were found to significantly interact with another and are tested together with their interaction term (FS:DP).

|  | Df | SumOf | R2 | F | Pr(>F) |  |
| --- | --- | --- | --- | --- | --- | --- |
| **metadata$Feedingstrategy** | **1** | **0.27959** | **0.12754** | 4.15 | 0.017 | * |
| metadata$Dietaryprotein | 1 | 0.0355 | 0.01619 | 0.5269 | 0.643 |  |
| metadata$SGR | 1 | 0.18034 | 0.08227 | 2.6769 | 0.076 | . |
| **metadata$HepGDH** | **1** | **0.24942** | **0.11378** | 3.7023 | 0.037 | * |
| metadata$IntestineGDH | 1 | 0.05476 | 0.02498 | 0.8129 | 0.423 |  |
| **metadata$FS:DP** | **1** | **0.24728** | **0.1128** | 3.6704 | 0.04 | * |
| Residual | 17 | 1.14529 | 0.52244 |  |  |  |
| Total | 23 | 2.19219 | 1 |  |  |  |

*Signif. codes: ‘*’ 0.05 ‘.’ 0.1*

**Table S2 PERMANOVA test on Weighted UniFrac dissimilarity values.**

Each factor tested as explanatory variable for the observed Weighted UniFrac dissimilarity values is shown with the degrees of freedom (Df), sum of squares (SumOf), R^2^-, F- and p-values. Factors that have a significant effect on the dissimilarity values are indicated in **bold**, with asterisks indicating the level of significance. Feeding strategy and Dietary protein were found to significantly interact with another and are tested together with their interaction term (FS:DP).

|  | Df | SumOfSqs | R2 | F | Pr(>F) |  |
| --- | --- | --- | --- | --- | --- | --- |
| metadata$Feedingstrategy | 1 | 0.0186 | 0.05907 | 1.7824 | 0.171 |  |
| metadata$Dietaryprotein | 1 | 0.005267 | 0.01673 | 0.5048 | 0.517 |  |
| metadata$SGR | 1 | 0.024421 | 0.07756 | 2.3403 | 0.127 |  |
| **metadata$HepGDH** | **1** | 0.042088 | **0.13366** | 4.0333 | 0.041 | * |
| metadata$IntestineGDH | 1 | 0.003921 | 0.01245 | 0.3757 | 0.606 |  |
| **metadata$FS:DP** | 1 | **0.043187** | 0.13715 | 4.1386 | 0.037 | * |
| Residual | 17 | 0.177397 | 0.56338 |  |  |  |
| Total | 23 | 0.31488 | 1 |  |  |  |

*Signif. codes: ‘*’ 0.05*

**Table S3 Comparison of the network properties of the microbial association networks of demand-fed and batch-fed carp gut microbiota.**

Statistical testing of the network properties was performed using permutation test (100 permutations) with the *NetCoMi* package in R. Significantly different properties are printed in **bold**.

| **Largest connected component (LCC)** | | | | |
| --- | --- | --- | --- | --- |
|  | Demand-fed | Batch-fed | abs.diff. | p-value |
| **Relative LCC size** | **0.183** | **0.817** | **0.633** | **0.009901** |
| Clustering coefficient | 0 | 0.075 | 0.075 | 0.49505 |
| **Modularity** | **0.435** | **0.672** | **0.237** | **0.019802** |
| Positive edge percentage | 100 | 96.61 | 3.39 | 0.683168 |
| **Edge density** | **0.182** | **0.05** | **0.132** | **0.019802** |
| **Natural connectivity** | **0.126** | **0.025** | **0.102** | **0.019802** |
| Vertex connectivity | 1 | 1 | 0 | 1 |
| Average dissimilarity* | 0.687 | 0.691 | 0.004 | 0.485149 |
| Average path length** | 2.022 | 3.883 | 1.861 | 0.079208 |
| **Whole network** | | | | |
|  | Demand-fed | Batch-fed | abs.diff. | p-value |
| **Number of components** | **24** | **12** | **12** | **0.039604** |
| Clustering coefficient | 0 | 0.075 | 0.075 | 0.445545 |
| **Modularity** | **0.805** | **0.672** | **0.133** | **0.039604** |
| Positive edge percentage | 94.872 | 96.61 | 1.738 | 0.821782 |
| **Edge density** | **0.022** | **0.033** | **0.011** | **0.019802** |
| **Natural connectivity** | **0.019** | **0.02** | **0.001** | **0.039604** |

*p-values: one-tailed test with null hypothesis diff=0*

**: Dissimilarity = 1 - edge weight*

***: Path length = Sum of dissimilarities along the path*

**Table S3-2: Jaccard index (similarity between sets of most central nodes)**

|  | Jacc | P(<=Jacc) | P(>=Jacc) |
| --- | --- | --- | --- |
| **degree** | **0.053** | **0.004736** | **0.999549** |
| **betweenness centr.** | **0** | **0.003425** | **1** |
| **closeness centr.** | **0.083** | **0.004871** | **0.999228** |
| **eigenvec. centr.** | **0.125** | **0.019900** | **0.995129** |
| hub taxa | 0 | 0.666667 | 1 |

*Jaccard index in [0,1] (1 indicates perfect agreement)*

**Table S3-3: Adjusted Rand index (similarity betw. clusterings)**

|  | wholeNet | LCC |
| --- | --- | --- |
| **ARI** | **0.134** | **0.049** |
| **p-value** | **0** | **0.001** |

*ARI in [-1,1] with ARI=1: perfect agreement betw. clusterings*

*ARI=0: expected for two random clusterings*

*p-value: permutation test (n=100) with null hypothesis ARI=0*
